# Assessment of drug allergy and determination of safe alternative medications: a retrospective observational study

**DOI:** 10.3389/fmed.2026.1913031

**Published:** 2026-08-17

**Authors:** Saltuk Buğra Kaya

**Affiliations:** Department of Chest Diseases, Division of Allergy and Clinical Immunology, Faculty of Medicine, Inönü University, Malatya, Türkiye

**Keywords:** anaphylaxis, beta-lactam antibiotics, drug hypersensitivity, drug provocation test (DPT), non-steroidal anti-inflammatory drugs (NSAIDs), safe alternative medications

## Abstract

**Background:**

Drug hypersensitivity reactions (DHRs) represent a major clinical challenge because of their heterogeneous presentations and their impact on future therapeutic choices. Accurate diagnosis and identification of safe alternative medications are essential to optimize patient management and avoid unnecessary drug restrictions.

**Methods:**

This retrospective observational study included 541 consecutive patients referred for evaluation of suspected drug hypersensitivity between January 2024 and June 2025. Demographic characteristics, comorbidities, clinical presentations, culprit drugs, and results of skin prick, intradermal, and oral provocation tests were analyzed. Drug classes, reaction phenotypes, and age-related differences were evaluated. Alternative medication challenge protocols were used to identify safe treatment options.

**Results:**

The study included 541 patients (70.1% female; mean age 43.9 ± 13.5 years). Non-steroidal anti-inflammatory drugs (NSAIDs) (36.0%) and beta-lactam antibiotics (22.7%) were the most frequently implicated drug classes. Urticaria and angioedema predominated in NSAID-related reactions, whereas beta-lactams and proton pump inhibitors (PPIs) were more commonly associated with anaphylaxis. The frequency of anaphylaxis increased with age and was highest among patients aged 55–64 years. Among 195 patients with isolated NSAID hypersensitivity, 119 (61.0%) demonstrated a cross-reactive pattern involving two or more NSAID groups. Celecoxib was tolerated by all 238 challenged patients with isolated NSAID hypersensitivity or combined NSAID and beta-lactam hypersensitivity. Among 167 patients with beta-lactam hypersensitivity, 104 underwent a standardized alternative non-beta-lactam antibiotic challenge protocol routinely implemented in our clinic, consisting of azithromycin, ciprofloxacin, metronidazole, clindamycin, tetracycline, and sulfamethoxazole–trimethoprim. No adverse reactions were observed with azithromycin or tetracycline, whereas systemic reactions occurred with ciprofloxacin, metronidazole, clindamycin, and sulfamethoxazole–trimethoprim. Tramadol induced urticaria/angioedema in a subset of patients with NSAID-induced or NSAID-exacerbated cutaneous disease but was tolerated by all patients with NSAID-exacerbated respiratory disease. Suspected local anesthetic allergies were not confirmed in any patient.

**Conclusions:**

NSAIDs and beta-lactam antibiotics were the leading causes of suspected drug hypersensitivity in this cohort. Systematic allergy evaluation, including skin testing and oral provocation, enabled accurate diagnosis and identification of safe therapeutic alternatives. Celecoxib emerged as a safe alternative for patients with NSAID hypersensitivity, while azithromycin and tetracycline were well tolerated among selected patients with beta-lactam allergy. These findings support the use of structured diagnostic assessment to improve patient safety, prevent unnecessary drug avoidance, and guide evidence-based selection of alternative therapies.

## Introduction

Drug hypersensitivity reactions (DHRs) encompass a wide spectrum of clinical manifestations, including urticaria, angioedema, anaphylaxis, and delayed-type cutaneous reactions, and constitute a major public health concern owing to their substantial impact on diagnostic accuracy, therapeutic decision-making, and healthcare systems. Global data indicate that the prevalence of self-reported drug allergy in the general population ranges between 5 and 15%; however, only a small proportion of these reports can be confirmed by diagnostic testing.

The most frequently implicated drug classes are non-steroidal anti-inflammatory drugs (NSAIDs) and beta-lactam antibiotics, with NSAIDs being responsible for a substantial proportion of all DHRs (1, 2). Nevertheless, clinical history alone is not always a reliable diagnostic tool; studies have shown that reported reactions are not consistent with true drug allergy in 30–60% of cases (3). Incorrect drug allergy labeling, particularly for antibiotics, restricts therapeutic options, often leads to unnecessary use of broad-spectrum alternatives, and increases healthcare costs (4). Therefore, skin prick and intradermal tests, as well as oral provocation tests—considered the gold standard—play a crucial role in establishing an accurate diagnosis and identifying safe alternatives (3–5).

Importantly, in clinical settings where allergy testing cannot be performed due to technical, logistical, or patient-related constraints, identifying safe alternative drugs becomes medically essential. Establishing such alternatives helps ensure effective treatment while minimizing the risk of hypersensitivity reactions.

Beta-lactam hypersensitivity has been reported in 1–10% of the general population, while studies in children have demonstrated a confirmed prevalence of 5.2% with direct oral provocation testing; severe reactions are exceedingly rare (0.04%) (6). Drug allergy registries have shown that NSAIDs and antibiotics are implicated at comparable frequencies in confirmed cases, with urticaria, angioedema, and respiratory symptoms being the most frequently observed clinical features.

In this context, evaluating the relationship between drug allergy diagnosis and demographic characteristics, reaction types, culprit drug classes, and comorbidities, as well as assessing the concordance between clinical history and diagnostic testing, and determining safe alternative drugs, are of great clinical importance. The current study aimed to investigate the characteristics of patients referred with a preliminary diagnosis of drug allergy, their culprit drugs, diagnostic test results, and associated comorbidities.

Consecutive patients referred to the Allergy and Immunology Clinic of a tertiary referral center for evaluation of suspected drug hypersensitivity reactions between January 2024 and June 2025 were retrospectively included in the study. A total of 541 patients who were referred for evaluation of suspected drug hypersensitivity reactions (DHRs) were included. Demographic characteristics, comorbidities, reaction features, and culprit drug classes were extracted from the institutional database. Reactions were classified as immediate (within 1 h of intake) or non-immediate and were further categorized into urticaria/angioedema, anaphylaxis, or delayed-type reactions.

Diagnostic evaluation included skin prick testing, intradermal testing, and, when clinically appropriate, drug provocation tests (DPTs). For patients with suspected immediate NSAID hypersensitivity, skin prick and intradermal tests were performed using non-irritating concentrations according to the recommendations of the EAACI Drug Allergy Interest Group (DAIG) practical guidance. Paracetamol was tested using the undiluted commercial preparation for skin prick testing and a maximum intradermal concentration of 0.1 mg/mL, while diclofenac was tested using the undiluted commercial preparation for skin prick testing. Dexketoprofen was tested using the undiluted commercial preparation for skin prick testing and a 1:10 dilution for intradermal testing, based on published non-irritating testing principles and our institutional protocol. As validated skin test concentrations are unavailable for most NSAIDs, including dexketoprofen, skin test findings were interpreted cautiously in conjunction with the patient's clinical history and, when appropriate, drug provocation testing. In patients with a history suggestive of severe immediate hypersensitivity, testing was initiated with serial dilutions (1:1,000, 1:100, 1:10, and the maximum non-irritating concentration) according to the patient's clinical history and risk assessment.

Positive skin test results were accepted only when no irritant response was observed in the negative control and were interpreted together with the clinical history and drug provocation test results when indicated.

Written informed consent was obtained before each DPT. Tests were performed under the direct supervision of an allergy specialist with full emergency preparedness. Baseline physical examination, peak expiratory flow (PEF), blood pressure, and heart rate were recorded before each dose. Testing was conducted between 09:00 and 12:00, with observation continuing until 17:00 for negative cases and until full recovery in positive cases. The dose interval was 30 min.

Positive skin test or drug provocation test results confirmed drug hypersensitivity, whereas patients with negative results underwent testing with alternative agents to identify safe therapeutic options. The study was approved by the Medical Ethics Committee of Inönü University (GO 2025/8382; September 30, 2025) and conducted in accordance with the Declaration of Helsinki. All participants provided written informed consent prior to inclusion, and patient confidentiality was strictly maintained throughout the study.

### Statistical analysis

Data were analyzed using IBM SPSS Statistics version 26.0 (IBM Corp., Armonk, NY, USA). Continuous variables were expressed as mean ± SD, and categorical data as frequencies and percentages. Normality was assessed by the Kolmogorov–Smirnov test. Group comparisons used the *t*-test or Mann–Whitney *U*-test, while categorical variables were evaluated with chi-square or Fisher's exact test.

Associations between drug classes and reaction phenotypes were analyzed using cross-tabulation and χ^2^ tests. Correlations between comorbidities and reaction severity were assessed by Spearman's coefficient. Age-related differences in anaphylaxis frequency and inconsistent allergy histories were also evaluated. A *p*-value < 0.05 was considered statistically significant.

## Results

A total of 541 patients (379 female, 70.1%; 162 male, 29.9%) with a mean age of 43.9 ± 13.5 years were included in the study. Among them, 312 had no comorbidities, 119 had chronic idiopathic urticaria, and 75 had asthma. At presentation, 86 patients (15.9%) reported symptoms not consistent with drug allergy; however, diagnostic testing was still performed. Of the 455 patients with a history of drug allergy, 106 (19.6%) presented with urticaria and/or angioedema, 336 (62.1%) with anaphylaxis, and 13 (2.4%) with delayed-type drug reactions.

The most frequently implicated drug classes were NSAIDs (36.0%) and beta-lactam antibiotics (22.7%), followed by combined NSAID and beta-lactam exposure (7.9%), quinolones (3.5%), and proton pump inhibitors (PPIs) (2.8%). The most commonly reported implicated individual drugs were dexketoprofen (116 patients), amoxicillin–clavulanate (85 patients), diclofenac (82 patients), paracetamol (33 patients), and acetylsalicylic acid (23 patients). NSAID exposure was significantly associated with urticaria/angioedema, whereas beta-lactam antibiotics and PPIs were more frequently associated with anaphylaxis, and quinolones with delayed-type reactions (*p* < 0.001). Urticaria/angioedema was most often observed in patients with chronic idiopathic urticaria, while anaphylaxis was more frequent in patients with asthma (*p* < 0.001). The demographic data, comorbidities, reaction types, and culprit drug groups of the patients are shown in Table 1.

**Table 1:** Clinical and demographic characteristics of patients with suspected drug hypersensitivity.

Characteristic	Male *N* (%)	Female *N* (%)	Total *N* (%)
Age (years) (mean ±*SD*)	44.89 ± 13.82	43.5 ± 13.43	
Comorbidities			
None	104 (33.3)	208 (66.6)	312 (57.6)
Chronic idiopathic urticaria	31 (26.0)	88 (74.0)	119 (22.0)
Asthma	20 (35.5)	55 (64.5)	75 (13.9)
Other	9 (25.7)	26 (74.3)	35 (6.5)
Types of drug reactions			
Symptoms and signs are not consistent with drug allergy	20 (22.7)	66 (77.3)	86 (15.6)
Urticaria and/or angioedema	30 (28.3)	76 (71.7)	106 (19.6)
Anaphylaxis	107 (31.8)	229 (68.2)	336 (62.1)
Delayed drugs reactions	5 (38.4)	8 (61.6)	13 (2.4)
The group of drugs that cause the reaction			
Isolated non-steroidal anti-inflammatory drug	71 (36.4)	124(63.6)	195 (36.0)
Isolated beta lactam group antibiotic	43 (34.9)	81 (65.1)	124 (22.7)
Drug allergy was not considered	16 (20)	64 (80)	80 (14.7)
Beta lactam antibiotic and non-steroidal anti-inflammatory drug	11 (25.5)	32 (74.5)	43 (7.9)
Quinolone group antibiotic	6 (31.5)	13 (68.5)	19 (3.5)
Proton pump inhibitor drugs	4 (26.6)	11 (73.4)	15 (2.7)
Local anesthetic drugs	2 (14.2)	12 (85.8)	14 (2.6)
General anesthetic drug	1 (10)	9 (90)	10 (1.8)

Age-stratified analysis revealed that inconsistent drug allergy histories were most common in the 18–24 age group and least frequent in the 55–64 age group (*p* < 0.001). However, the distribution of drug classes did not differ significantly across age groups. In the geriatric subgroup, NSAIDs and beta-lactam antibiotics were the leading causes of drug allergy. Among 39 elderly patients, 4 had non-allergic complaints, while of the remaining 35, 19 were allergic to NSAIDs, 8 to beta-lactams, and 3 to both. The frequency of anaphylaxis was lowest in patients younger than 25 years (45.8%) and highest in those aged 55–64 years (75.1%). The distribution of drug hypersensitivity reactions by drug class, diagnostic outcomes, and identified safe alternatives are shown in Table 2.

**Table 2:** Distribution of reaction types and clinical characteristics according to age groups.

Age range	Predominant reaction type(s)	Statistical significance (*p*)	Clinical interpretation
Comorbidities and reaction	Chronic idiopathic urticaria	*p* < 0.001	Urticaria/angioedema more frequent in urticaria
	Asthma	*p* < 0.001	Anaphylaxis more frequent in asthma
Reaction consistency	Consistent: 455 (84.1%) Non-consistent: 86 (15.9%)	Age group differences: *p* < 0.001	Inconsistent drug allergy histories were most frequently observed in the 18–24 age group and least common in the 55–64 age group

Diagnostic testing was performed in 89 patients (16.4%) using skin prick and/or intradermal test methods, with the highest positivity rates observed for dexketoprofen (*n* = 18), paracetamol (*n* = 15), ciprofloxacin (*n* = 12), and pantoprazole (*n* = 12). Oral provocation testing induced early-type reactions in 62 patients (11.4%), most frequently triggered by acetylsalicylic acid (*n* = 19), tramadol (*n* = 11), ciprofloxacin (*n* = 5), and amoxicillin–clavulanate (*n* = 5).

Among patients with isolated NSAID hypersensitivity, excluding those with concomitant beta-lactam allergy (*n* = 195), 119 reported reactions to at least two different NSAID groups, suggesting a cross-reactive hypersensitivity pattern. Dexketoprofen (*n* = 116) and diclofenac (*n* = 82) were the most commonly implicated agents, while paracetamol (*n* = 33), acetylsalicylic acid (*n* = 23), and ibuprofen (*n* = 20) were also frequently reported. Less common agents included flurbiprofen (*n* = 18), etodolac (*n* = 9), naproxen (*n* = 2), meloxicam (*n* = 1), nimesulide (*n* = 1), and metamizole (*n* = 1).

Of the NSAID-hypersensitive patients, 31 had NSAID-induced cutaneous disease, 43 had NSAID-exacerbated cutaneous disease, 38 had NSAID-exacerbated airway disease, and 7 had single-agent-induced cutaneous disease. Among the study population, 195 patients had isolated NSAID hypersensitivity, while an additional 43 patients had combined NSAID and beta-lactam hypersensitivity, resulting in a total of 238 patients eligible for celecoxib provocation testing. All 238 patients underwent oral provocation with celecoxib, and no hypersensitivity reactions were observed, corresponding to a 100% tolerance rate. These findings support celecoxib as a safe alternative analgesic in patients with both isolated and combined NSAID hypersensitivity. All 112 patients with NSAID-induced skin disease, NSAID-exacerbated skin disease, and NSAID-exacerbated respiratory disease underwent oral challenge testing with tramadol. Tramadol provoked urticaria/angioedema in 11 patients with NSAID-induced or NSAID-exacerbated skin disease; however, no reactions occurred in patients with NSAID-exacerbated respiratory disease.

Among the 167 patients with beta-lactam hypersensitivity, 104 underwent a standardized alternative antibiotic challenge protocol routinely implemented in our clinic to identify safe therapeutic options. All 104 patients underwent oral provocation testing with azithromycin, ciprofloxacin, metronidazole, clindamycin, tetracycline, and sulfamethoxazole–trimethoprim. Systemic reactions occurred in 6 of 104 patients (5.8%) challenged with ciprofloxacin, 2 of 104 (1.9%) with metronidazole, 1 of 104 (1.0%) with clindamycin, and 4 of 104 (3.8%) with sulfamethoxazole–trimethoprim. In contrast, no adverse reactions were observed during azithromycin or tetracycline provocation testing (0/104 for both agents), supporting their safety as alternative treatment options in this cohort.

Proton pump inhibitor allergy was identified in 15 patients, 12 of whom demonstrated positive skin tests to pantoprazole, the suspected culprit agent. All affected patients subsequently tolerated oral administration of the H_2_-receptor antagonist famotidine, supporting its safety as an alternative treatment option.

Among 14 patients with suspected local anesthetic allergy (seven to articaine hydrochloride and seven to lidocaine hydrochloride), all reactions were consistent with side effects rather than true allergy. All patients had negative skin test results followed by negative subcutaneous drug provocation tests, confirming tolerance to the evaluated local anesthetics.

Perioperative anaphylaxis was assessed in 10 patients. Six patients were allergic to general anesthetics, two to latex, and two had negative test results. Within the general anesthetic group, three patients were sensitized to rocuronium, two to both ketamine and midazolam, and one to midazolam alone.

Finally, eight patients were evaluated for contrast media-induced anaphylaxis, and all diagnostic tests were negative. The distribution of drug hypersensitivity reactions by drug class, diagnostic outcomes, and identified safe alternatives are shown in Table 3.

**Table 3:** Distribution of drug hypersensitivity reactions by drug class, diagnostic outcomes, and identified safe alternatives.

Drug class/agent	*n* (%)	Predominant reaction type(s)	Key diagnostic findings (*N*)	Safe alternatives
Isolated and combined NSAID	238 (44.0)	Urticaria/angioedema, airway disease	**Skin test:** dexketoprofen (18), diclofenac (82), paracetamol (33) **Oral provocation:** ASA (19), tramadol (11)	Celecoxib (all tested negative N:238)
Beta-lactams	167 (30.9)	Mainly anaphylaxis	Amoxicillin-clavulanate (85) **Oral provocation:** amoxicillin–clavulanate (5), alternative antibiotic challenges (*n* = 104 each): ciprofloxacin (6), metronidazole (2), clindamycin (1), sulfamethoxazole–trimethoprim (4), azithromycin (0), tetracycline (0)	Azithromycin and Tetracycline (confirmed tolerated alternatives)
PPIs	15 (2.8)	Anaphylaxis	**Skin test:** pantoprazole (12/12 positive)	Famotidine (all tests negative)
Quinolones	19 (3.5)	Delayed-type reactions	**Skin test:** ciprofloxacin (12) **Oral provocation:** ciprofloxacin (5)	Avoidance of culprit drugs
Perioperative agents	10	Anaphylaxis	Rocuronium (3), ketamine (2), midazolam (3), latex (2)	Avoidance of culprit drugs
Local anesthetics	14	Side effects, not true allergy	All skin tests negative; all subcutaneous provocation tests were negative	Re-evaluation for drug side effects
Contrast media (ICM)	8	Suspected anaphylaxis	All skin tests negative	Most immediate hypersensitivity reactions likely non-IgE-mediated

The NSAID category includes 195 patients with isolated NSAID hypersensitivity. Celecoxib oral provocation testing was performed in a total of 238 patients, comprising 195 patients with isolated NSAID hypersensitivity and an additional 43 patients with combined NSAID and beta-lactam hypersensitivity. All patients tolerated celecoxib.

## Discussions

In our cohort, the frequency of anaphylaxis varied across age groups, with the lowest rate in patients under 25 years (45.8%) and the highest in those aged 55–64 years (75.1%). This finding suggests that increasing age may be associated with a greater risk of severe DHRs. Older patients are more likely to have multiple comorbidities, polypharmacy, and repeated exposures to medications, which may contribute to this elevated risk. Conversely, the relatively lower frequency in younger individuals may reflect fewer cumulative drug exposures and a lower prevalence of chronic diseases requiring treatment. These findings are consistent with previous reports indicating that age is an important factor influencing both the incidence and severity of drug-induced anaphylaxis.

Our findings align with the established classification of NSAID hypersensitivity, which differentiates between cross-reactive and single-drug-induced mechanisms. In our cohort, NSAIDs were the most frequently implicated drugs, accounting for 36.0% of reported hypersensitivity reactions. Most reactions manifested as urticaria and/or angioedema, particularly in patients with chronic idiopathic urticaria, consistent with the cross-reactive phenotype in which COX-1 inhibition triggers inflammatory cell activation and mediator release. Among 195 patients with isolated NSAID hypersensitivity, 119 (61.0%) experienced reactions to two or more different NSAID groups, consistent with a cross-reactive hypersensitivity phenotype. Dexketoprofen and diclofenac were the most commonly implicated agents, reflecting local prescribing habits, while paracetamol was implicated in 33 cases, suggesting that even weak COX-1 inhibitors may provoke reactions in highly sensitive individuals.

Single-drug reactions, likely mediated by IgE, were less common but included isolated cases involving meloxicam, nimesulide, and metamizole. All 112 patients with NSAID-induced skin or airway disease tolerated selective COX-2 inhibition with celecoxib, confirming the safety of this agent in cross-reactive phenotypes. These results highlight the importance of integrating the type of reaction, the patient's comorbidities, and the pharmacological properties of the responsible drug. Careful clinical evaluation and confirmatory provocation testing are essential to distinguish cross-reactive sensitization from single-drug sensitization, with direct implications for safe treatment planning (7–11).

While opioids and their synthetic derivatives, particularly tramadol, are widely prescribed for pain management, hypersensitivity reactions remain a concern. Most opioid reactions are pseudoallergic, mediated directly by mast cell and basophil activation, but true IgE-mediated mechanisms are rare. Both mechanisms may present as angioedema. Tramadol-induced reactions are considered uncommon, with an estimated incidence ranging from 1 in 1,000 to 1 in 10,000 patients. Consistent with previous reports, including the study by Asero et al., which demonstrated that 18% of chronic urticaria patients with NSAID hypersensitivity are intolerant to tramadol, we observed that tramadol induced urticaria/angioedema in 11 patients (9.8%), while patients with NSAID-exacerbated respiratory disease tolerated tramadol without reactions. These findings suggest that tramadol is not a universally safe alternative for NSAID-exacerbated skin disease and should only be considered after individualized diagnostic testing (12–14).

Beta-lactam antibiotics were the second most frequently implicated drug class, reported in 22.7% of patients, with amoxicillin–clavulanate being the most common single agent. Among patients with a history of beta-lactam hypersensitivity, anaphylaxis was the predominant clinical presentation. Oral challenge confirmed early-type reactions in some patients, while skin test positivity was less frequent, consistent with existing literature. In addition, 104 patients underwent oral challenge with alternative antibiotics, including azithromycin, ciprofloxacin, metronidazole, clindamycin, sulfamethoxazole–trimethoprim, and tetracycline. Systemic reactions occurred with ciprofloxacin, metronidazole, clindamycin, and sulfamethoxazole–trimethoprim. Notably, although azithromycin and tetracycline also belonged to non-beta-lactam antibiotic classes and were included in the same alternative antibiotic challenge protocol, no adverse reactions were observed with either agent. These findings underscore the clinical burden of beta-lactam allergy labels and the risks of *de novo* hypersensitivity to alternative agents.

Although penicillins are widely prescribed, they remain among the most frequently reported causes of drug allergy, with unconfirmed allergy labels affecting 2–13% of the population. Evidence indicates that approximately 90% of patients labeled as penicillin-allergic are not truly allergic, as demonstrated by systematic allergy and drug testing. In contrast to the general literature, our findings show that beta-lactams were the drug class most commonly responsible for anaphylaxis in our cohort, likely reflecting a referral bias toward severe cases in allergist consultations. These results underscore the importance of systematic allergy assessment to prevent unnecessary avoidance of first-line antibiotics and to inform the safe selection of alternatives (15–17).

Proton pump inhibitors (PPIs) are widely prescribed worldwide, yet hypersensitivity reactions, including anaphylaxis, are increasingly recognized. In our series, 15 patients had a history of PPI-induced anaphylaxis, and all 12 who underwent skin testing with pantoprazole tested positive, consistent with previous studies reporting high sensitivity of skin tests in PPI allergy (18). Given the high cross-reactivity among PPIs, all patients underwent an oral challenge with the H2-receptor antagonist famotidine, which was well tolerated. These results are in line with earlier reports showing cross-reactivity between PPIs but not between PPIs and H2-receptor antagonists, supporting the role of H2-blockers as safe therapeutic alternatives when acid suppression is required.

Adverse reactions to local anesthetics (LAs) are most often attributable to non-allergic mechanisms such as vasovagal syncope, anxiety, or pharmacological effects of epinephrine (19). True hypersensitivity to LAs is rare, accounting for fewer than 1% of all reported cases (20). In line with this evidence, all patients in our cohort who presented with suspected LA allergy had negative skin and provocation tests, confirming tolerance and excluding true hypersensitivity. These findings emphasize the importance of careful evaluation to distinguish side effects from allergy and prevent unnecessary drug avoidance.

Approximately 60% of hypersensitivity reactions occurring during anesthesia are IgE-mediated. Large French studies involving nearly 4,000 perioperative anaphylaxis cases identified muscle relaxants (62%), latex (16.5%), hypnotics (7.4%), antibiotics (4.7%), plasma substitutes (3.6%), and opioids (1.9%) as the leading culprits (21). Our findings are consistent with these data: among six patients with general anesthetic allergy, three were sensitized to rocuronium, two to ketamine and midazolam, and one to midazolam alone. In addition, two patients were allergic to latex. These results reinforce the pivotal role of neuromuscular blocking agents in perioperative anaphylaxis while also highlighting the contribution of sedative agents and latex (22).

Finally, hypersensitivity reactions to iodinated contrast media (ICM) continue to pose a significant clinical challenge. These reactions can be classified into immediate and non-immediate types, with immediate reactions occurring within 1 h of exposure and often mediated by IgE, while non-IgE-mediated mechanisms, including direct mast cell or basophil activation, complement activation, or idiosyncratic responses, are responsible for a substantial proportion of both immediate and delayed reactions. Skin testing remains the cornerstone for diagnosing ICM allergy, though its sensitivity varies widely—from 4.2% to 73%—depending on reaction severity, timing, and test modality. A 2015 meta-analysis reported pooled positivity rates of 17% overall and 52% in severe immediate reactions (23). In our series, eight patients were evaluated for suspected ICM-induced anaphylaxis, yet all tests were negative, emphasizing the limitations of current diagnostic tools and the need to recognize non-IgE-mediated pathways when managing suspected contrast reactions.

In conclusion, this study delineates the heterogeneous clinical spectrum of drug hypersensitivity reactions (DHRs) and underscores the necessity of systematic evaluation through skin testing and oral challenge procedures. NSAIDs and beta-lactam antibiotics were identified as the most frequently implicated agents, with anaphylaxis occurring more commonly in older patients. Accurate distinction between cross-reactive and selective hypersensitivity mechanisms is essential for establishing an accurate diagnosis and ensuring safe therapeutic decision-making.

Among patients with isolated NSAID hypersensitivity or combined NSAID and beta-lactam hypersensitivity, celecoxib was tolerated by all 238 challenged patients, supporting its role as a safe alternative analgesic. Similarly, among patients with beta-lactam hypersensitivity, azithromycin and tetracycline were tolerated without adverse reactions during standardized alternative antibiotic challenge testing, supporting their use as reliable therapeutic alternatives. In contrast, systemic reactions were observed with ciprofloxacin, metronidazole, clindamycin, and sulfamethoxazole–trimethoprim, highlighting the need for individualized evaluation before prescribing alternative antibiotics.

These findings emphasize the importance of targeted allergy assessment to optimize clinical management, prevent unnecessary avoidance of first-line medications, and facilitate the identification of safe and effective therapeutic alternatives in patients with DHRs.

## Limitation

This study has several limitations. First, its retrospective design may have introduced inherent selection and information biases. Second, because the study was conducted at a tertiary allergy referral center, the study population likely represents patients with more severe or complex drug hypersensitivity reactions. This referral bias may explain the relatively high proportion of anaphylaxis observed in our cohort and may limit the generalizability of our findings to primary care or general hospital settings. Nevertheless, the large sample size and the systematic diagnostic evaluation performed in a specialized allergy center strengthen the clinical relevance of our findings.

## Data Availability

The raw data supporting the conclusions of this article will be made available by the authors, without undue reservation.
